# Astaxanthin-Based Biomaterials for Tissue Repair and Drug Delivery Systems

**DOI:** 10.34133/bmr.0282

**Published:** 2025-12-03

**Authors:** Yibing Wang, Huaqian Xue, Chuchu Sun, Qiancheng Gu, Liang Chen, Zhengqiu Lin, Liyuan Xu, Lanjie Lei, Qiujie Li, Zhangwei Zhao

**Affiliations:** ^1^ Key Laboratory of Artificial Organs and Computational Medicine of Zhejiang Province, Shulan International Medical College, Institute of Translational Medicine, Zhejiang Shuren University, Hangzhou, Zhejiang 310015, China.; ^2^ The Third Affiliated Hospital of Wenzhou Medical University, Wenzhou 325200, China.

## Abstract

Astaxanthin (AST), a potent bioactive compound known for its exceptional antioxidant, anti-inflammatory, and anti-apoptotic capacities, has been widely applied in advanced biomedical domains, including regenerative tissue engineering and targeted drug delivery systems. However, its chemical instability limits broader applications. To address this issue, various multifunctional biomaterials, such as nanoliposomes, nanoparticles, glass microspheres, and algal calcium beads, have been employed to stabilize AST and enhance its therapeutic efficacy. This review provides a comprehensive overview of AST, examines its mechanisms of action, and discusses the development and biomedical applications of AST-based biomaterials. We demonstrate the excellent properties and potential applications of these biomaterials in various biomedical contexts, outline existing challenges, and propose future directions to optimize their design and advance their clinical translation.

## Introduction

Astaxanthin (AST) [1] is a member of the ketone carotenoid family and is mainly found in marine organisms such as salmonidae, krill, lobsters, and red algae [2], and demonstrates antioxidative [3,4], anti-inflammatory [5], and anti-apoptotic properties [1]. The structural uniqueness of AST, particularly its extended conjugated system and terminal carbonyl moieties, enables the effective neutralization of reactive oxygen species (ROS) via both physical quenching and chemical redox reactions, forming the basis of its antioxidant mechanism [6]. For instance, AST can reduce the damage to muscle cells caused by free radicals generated during exercise and promote the repair and regeneration of muscles. Its antioxidant effect can also prevent the destruction of skin collagen and elastic fibers, keeping the skin firm and smooth for anti-aging applications [7]. The anti-inflammatory effect of AST reduces the inflammatory response in the eyes, improves ocular blood circulation, and maintains eye health [8]. In the eyes, AST can effectively filter blue light and thus reduce damage to retinal cells and prevent retinal diseases. In addition, AST plays an important role in inhibiting the oxidation of low-density lipoprotein cholesterol in the bloodstream, thereby contributing to a reduced risk of atherosclerosis.

Biomaterials are specific functional materials, either occurring naturally or being artificially synthesized, that can be in contact with and interact with biological systems. They can be used for the treatment, replacement, repair, or induction of regeneration of cells, tissues, and organs. AST can be carried by 3 major categories of biomaterials: natural biomaterials [9], synthetic biomaterials, and bio-derived materials. Natural biomaterials, such as collagen, chitosan (COS), and cellulose, are directly obtained from biological organisms; synthetic biomaterials are prepared through chemical synthesis or other artificial methods based on polylactic acid (PLA), polyglycolic acid, and their copolymers, such as polylactic-co-glycolic acid (PLGA); bio-derived materials, which combine the biological activity of natural materials with the specific properties of artificial materials, are obtained by chemically modifying and processing natural materials.

AST, as an efficient natural antioxidant, has limited applicability in disease treatment owing to issues such as low bioavailability, poor stability, and difficulty in precise delivery. Through innovations in dosage forms and the introduction of new biomaterial carriers in recent years, the administration methods of AST have been optimized, improving its therapeutic effects in specific diseases [10,11]. Traditional dosage forms in oral administration, such as oil solutions or solid dispersions in capsules and tablets, although convenient to take, suffer from low absorption rates owing to the destruction of AST in the gastrointestinal environment, with absorption rates less than 10%. To address this limitation, researchers have developed biomaterial-based nano-drug delivery systems. For instance, Zhu et al. [12] encapsulated AST in polyethylene glycol-grafted chitosan (PEG-g-CS) nanoparticles and produced oral nano-formulations via the solvent evaporation method. This dosage form not only improved the water solubility and intestinal absorption efficiency of AST but also significantly enhanced its oral bioavailability; this provides a more effective oral delivery route for treating oxidative stress-related intestinal or systemic diseases. In the treatment of tumors, design of the dosage form mostly focuses on targeting and intracellular delivery effects. Haung et al. [13] constructed an oil-in-water AST nano-emulsion using d-α-tocopheryl polyethylene glycol succinate as an emulsifier. After intravenous injection, this nano-emulsion effectively induced apoptosis in melanoma cells and inhibited lung metastasis, demonstrating the potential value of dosage form improvements in the treatment of malignant tumors. Additionally, in local and precise administration, biomaterial carriers such as nanoparticles, liposomes, and polymer micelles have been used in the development of ocular drug delivery systems. These dosage forms can overcome the physiological barriers of the eye and achieve effective enrichment of AST in ocular tissues, providing new therapeutic avenues for eye diseases such as age-related macular degeneration (AMD) [14]. In summary, by encapsulating AST in functionalized biomaterials to construct nanoparticles, nano-emulsions, liposomes, and other new dosage forms, the limitations of traditional administration have been overcome and targeted tissue delivery, controlled release effects, and therapeutic efficacy have been achieved. This opens up new avenues for the application of AST in drug delivery and tissue engineering.

This review provides an overview of AST-based biomaterials and their applications in biomedicine [15]. Their current development status across various biomedical fields is summarized. Furthermore, by analyzing the problems encountered in current developments, future research directions are suggested, such as exploring more effective composite biomaterials and optimizing preparation processes, to promote in-depth development in this field.

## Overview and Mechanisms of Action of AST

**Fig. 1.**
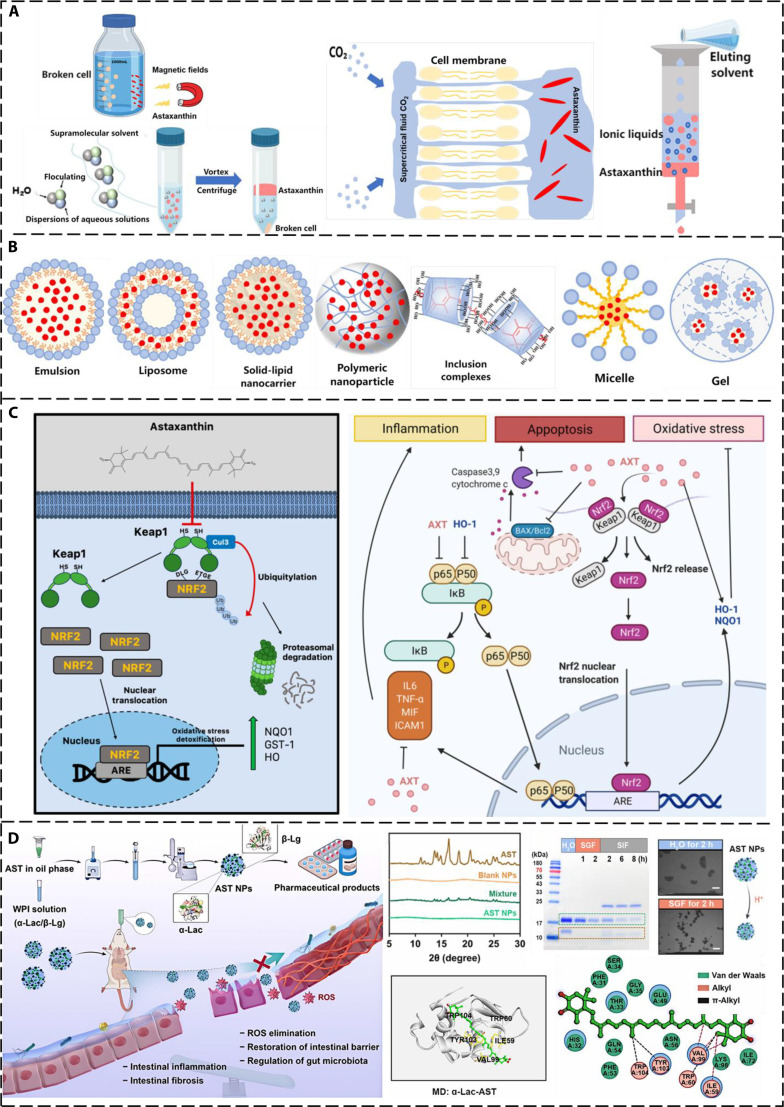
The extraction method, delivery strategy, mechanism of action, and application of AST. (A) Four main extraction techniques [17]. (B) Different biological materials for delivering AST [20]. (C) Localization of AST in the cell membrane promoting cell proliferation via activation of forkhead box O3 (FOXO3), phosphoinositide 3-kinase (PI3K), and mitogen-activated protein kinase kinase (MEK) signaling pathways; antioxidant and anti-inflammatory effects achieved through its interaction with these signaling pathways [21,22]. (D) Schematic representation of AST-NP formulation process, highlighting dual mechanisms involving inflammatory pathway inhibition and fibrotic process suppression [52].

AST is derived from various microorganisms and marine animals [16], including liposoluble red-orange pigments from bacteria, yeasts, fungi, microalgae, shrimp, and crabs. *Haematococcus pluvialis* is regarded as the most abundant natural source of AST. As a lipophilic compound [17], AST belongs to the ketone-type carotenoid family and can dissolve in organic solvents and oils, but not in water. Its chemical structure [18] is composed of 2 terminal rings connected by a polyethylene chain. This molecule contains 2 asymmetric centers at positions 3 and 3′ of the B-ion ring with a hydroxyl group (-OH) at both ends. The 13 conjugated double bonds in the AST molecule extend throughout its structure, conferring powerful antioxidant properties [19], with an efficacy approximately 6,000 times greater than that of vitamin C. However, owing to the high reactivity of these double bonds, the special structure is susceptible to environmental factors, undergoing oxidation, cleavage, and isomerization reactions, which lead to the loss of the biological activity of AST. AST can cross the bilayer structure of cell membranes to protect the components both inside and outside the cells. Common methods for extracting AST include organic solvent extraction, ion liquid-mediated extraction, supramolecular solvent extraction, and magnetic nanoparticles-assisted extraction (Fig. 1A). These methods involve crushing raw materials [17], such as shrimp shells and algae, soaking them in organic solvents, filtering them to remove residues, and evaporating the solvents to obtain crude AST extracts. In an enzymatic hydrolysis method, the crude extracts are mixed with enzyme solutions, and reactions occur under suitable conditions to break down cell walls and lipids. Then, AST is separated through centrifugation or filtration. Although current applications of AST are limited by its poor stability, low bioavailability, and high production cost, researchers have adopted various functional biomaterials to enhance the solubility [20], stability, and targeting properties of AST, which is expected to overcome these limitations and expand its application scope (Fig. 1B).

As research progresses, AST has demonstrated significant effects in antioxidation, anti-inflammation, anti-apoptosis, and metabolic modulation because it can regulate multiple signaling pathways and molecular targets. This provides a theoretical basis for its clinical application to treat various diseases, such as neurodegenerative diseases, cardiovascular diseases, and metabolic syndromes. Moreover, by studying the molecular targets and signaling pathways of AST, the drug design and dosage forms can be optimized. For example, AST enhances the antioxidant performance of cells by activating the nuclear factor erythroid 2-related factor 2 (Nrf2)/antioxidant response element (ARE) signaling pathway. ARE is a DNA motif located in the gene promoter area. It has the ability to bind with the Nrf2 transcriptional regulator and initiate the transcription of downstream genes involved in antioxidant activities. Nrf2 is a pivotal transcriptional regulator in the ARE pathway, which promotes nuclear translocation. The activated Nrf2/ARE pathway increases the production of antioxidant enzymes [such as superoxide dismutase (SOD) and heme oxygenase-1 (HO-1)], effectively eliminating ROS and reducing oxidative stress-mediated cellular damage (Fig. 1C) [21,22]. Furthermore, the anti-inflammatory mechanism of AST is attributed to its inhibitory effect on the nuclear factor κ-light-chain-enhancer of activated B cells (NF-κB) pathway. NF-κB is a pivotal transcriptional regulator that governs the expression of pro-inflammatory mediators, such as tumor necrosis factor-α (TNF-α), interleukin-6 (IL-6), and IL-1β [23]. AST remarkably mitigates inflammatory reactions by suppressing the nuclear translocation of NF-κB and the production of downstream pro-inflammatory mediators [24]. This mechanism holds paramount significance in the management of chronic inflammatory disorders, including rheumatoid arthritis and atherosclerotic vascular disease. In addition, the Nrf2/ARE and Keap1 pathways alleviate oxidative damage by enhancing antioxidant defense capabilities. Pathways such as NF-κB, Toll-like receptor 4 (TLR4)/myeloid differentiation primary response 88 (MyD88), and cyclooxygenase-2 (COX-2)/inducible nitric oxide synthase (iNOS) are mainly involved in inhibiting the production and release of inflammatory factors; pathways such as mitogen-activated protein kinase (MAPK) [25], phosphoinositide 3-kinase (PI3K)/Akt/mTOR, and Bcl-2/Bax affect cell survival and autophagy by regulating kinase activity and the balance of apoptosis-related proteins [26,27]. SIRT1 [28] and PPAR-γ play a role in metabolic regulation and anti-aging [29]. The inhibition of matrix metalloproteinase (MMP) helps maintain the stability of the extracellular matrix (ECM). Mitochondria, as the core organelle, not only participate in energy synthesis but also play a key role in ROS clearance and anti-apoptosis [30,31]. These pathways together form a multi-target and multi-level network of action, which is of great significance in maintaining cellular homeostasis and resisting pathological damage [32–35]. This review summarizes the different signaling pathways and molecular targets through which AST exerts anti-inflammatory and antioxidant effects in Table 1.

**Table 1.** Molecular targets, modes of action, and physiological effects of astaxanthin

Molecular targets/pathways	Mode of action	Effectiveness of the action	References
Nrf2/ARE	Activate Nrf2 and promote the expression of antioxidant enzymes	Reduce oxidative stress and protect cells from ROS damage.	[21]
NF-κB	Inhibition of NF-κB nuclear translocation and downstream inflammatory factors	Play an anti-inflammatory role and relieve chronic inflammation	[24]
Mitogen-activated protein kinase (MAPK)	Inhibit phosphorylation of c-Jun N-terminal kinase (JNK), extracellular signal-regulated kinase (ERK), and p38 mitogen-activated protein kinase (p38 MAPK)	Alleviate cell apoptosis and inflammation	[25]
Phosphoinositide 3-kinase (PI3K)/protein kinase B (Akt)/mechanistic target of rapamycin (mTOR)	Activate Akt and inhibit excessive activation of mTOR	Inhibit excessive autophagy and apoptosis and improve the survival rate of cells	[26,27]
SIRT1	Up-regulate the expression of SIRT1 and enhance the deacetylation activity	Delay aging and improve metabolic syndrome	[28]
B cell lymphoma 2 (Bcl-2)/Bcl-2-associated X protein (Bax) family	It up-regulate the anti-apoptotic protein Bcl-2 and down-regulate the anti-apoptotic protein Bax	Inhibit mitochondrial pathway apoptosis, protect neurons and cardiomyocytes	[29]
Mitochondria	By embedding into the inner mitochondrial membrane, it directly neutralizes ROS, reducing oxidative damage from the source. It can also protect the electron transport chain, maintain membrane potential and ATP synthesis, and increase the energy yield of cells.	Reduce oxidative damage, resist apoptosis, and protect neurons and cardiomyocytes. Effectively delay the aging process related to mitochondrial dysfunction.	[30]
Matrix metalloproteinase (MMP)	Inhibit the activity of MMP-2/MMP-9	Prevent extracellular matrix degradation, inhibit tumor metastasis and rupture of atherosclerotic plaques	[31]
Toll-like receptor 4 (TLR4)/myeloid differentiation primary response 88 (MyD88)	Inhibition of the interaction between TLR4 and MyD88 inhibits downstream inflammatory signaling	Reduce the inflammatory response induced by endotoxin	[32]
Cyclooxygenase-2 (COX-2)/inducible nitric oxide synthase (iNOS)	Inhibit the expression of COX-2 and iNOS	Reduce the excessive production of prostaglandins and nitric oxide, and alleviate inflammation.	[33]
Peroxisome proliferator-activated receptor γ (PPAR-γ)	Activate the PPAR-γ receptor	Regulate lipid metabolism and improve insulin sensitivity	[34]
Kelch-like ECH-associated protein 1 (Keap1)	Bind to Keap1 and release Nrf2 into the nucleus	Enhance the antioxidant defense system	[35]

## Biomaterials for AST Loading

### Liposomes

#### Preparation of liposomes loaded with AST

At present, there are various biomaterials capable of delivering AST. We have summarized their characteristics and applications as shown in Table 2. The thin-film hydration method [36] is a common method to prepare liposomes loaded with AST. First, AST and phospholipids are dissolved in ethanol. The reaction mixture is subsequently transferred to a rotavapor, and the solvent is removed under vacuum conditions to generate a homogeneous dried lipid film on the interior surface of the vessel. Water or a buffer solution is added to the container to hydrate the film, producing multilayer liposomes with encapsulated AST, which are then subjected to ultrasonic treatment to obtain small, single-layer AST liposomes.

**Table 2.** Summary of the characteristics and applications of astaxanthin drug delivery systems

Material category	Material advantages	Material drawbacks	Therapeutic field	References
Liposome	Its unique phospholipid bilayer effectively enhances the bioavailability of AST, achieving sustained, controllable release and targeted delivery of AST	Poor stability, low encapsulation rate, prone to leakage, and high material cost	Repairs liver damage caused by alcohol or toxic substances, and resists the adverse effects of ionizing radiation on the skin	[43]
Nanoparticles	Improve the stability of astaxanthin, enhance its solubility, and achieve precise targeting and controlled release	The preparation process involves loading, with a limited drug loading capacity and the toxicity being uncertain.	Inhibit the metastasis of cancer cells and promote wound healing	[52]
Self- nanoparticulate emulsified drug delivery system (SNEDDS)	Significantly improved the stability and antioxidant properties of astaxanthin, enhanced its absorption, and exhibited excellent physical stability	Lack of active targeting ability and limited drug loading capacity	Neurological disorders, cardiovascular diseases, resistance to skin aging caused by ultraviolet rays	[53]
3D-printed dressings	With outstanding biological activity, antibacterial properties, the ability to promote tissue repair, and stability	The stability is uncertain, there is a risk of toxicity, and the cost is high.	Promote wound healing	[55]
Microcapsule	As a solid barrier, it protects astaxanthin, enhancing its bioavailability, and enabling targeted delivery and precise controlled release	The particle size is relatively large, and the ratio of the wall material may lead to instability of the structure	Realizing precise targeted delivery for gastrointestinal diseases	[57]
Polymerized micelles	The problem of poor water solubility of astaxanthin has been solved, effectively enhancing the drug-carrying capacity, targeting ability, and stability of astaxanthin.	The controlled- release effect is not satisfactory. Limitations in the selection of carrier materials and limited drug loading capacity.	Promote the proliferation of mesenchymal stem cells	[58]
β-Cyclodextrin (CD)	Provides extremely strong stability protection for astaxanthin, with high safety and a relatively simple preparation process	Limited drug loading capacity and lack of targeting properties	Has a significant protective effect against liver cell damage and enhances immunity	[60]

#### Liposomes loaded with AST

Liposomes are closed vesicles composed of a phospholipid bilayer, usually made up of natural or synthetic phospholipids that can encapsulate water- or lipid-soluble substances. Liposomes demonstrate biodegradability, biocompatibility, low toxicity, and sustained-release properties, making them well suited for drug delivery systems [37]. Employing liposomes as a biocompatible material to load AST can efficiently address the limitations of AST [38]. Researchers developed nano-liposomes with high AST encapsulation efficiency, which not only enhanced the bioavailability of AST but also improved its stability and water dispersibility [39], supporting the continuous release of AST. Wu et al. [40] developed Asta-lipo for the treatment of alcohol-related liver pathologies. The preparation method of Asta-lipo involved 4 steps, i.e., the preparation of a phospholipid 1,2-distearoyl-sn-glycero-3-phosphocholine lipid film, hydration, homogenization, and purification. Further studies showed that Asta-lipo repaired liver damage caused by alcohol or toxic substances through the intraperitoneal route, which was proven by the detection of alanine aminotransferase. Furthermore, Vu et al. [41] developed a nanostructured lipid carrier (ASX-NLC) using a combined method consisting of thermal homogenization and ultrasonic treatment. The delivery vehicle efficiently augmented the stability, dispersibility, and biopharmaceutical availability of AST, facilitating its sustained release and site-specific delivery. In vitro experimental results [42] indicated that, at the optimal concentration of AST, ATX-NLC effectively reduced cell death after x-ray irradiation relative to the control group. Therefore, ATX-NLC has the potential to resist the adverse effects of ionizing radiation on the skin during radiotherapy. Extracellular nanovesicles (ENVs) have been employed as carriers for AST due to their favorable biocompatibility, low immunogenicity, intrinsic cytocompatibility, precise targeting capacity, and ability to permeate across vascular endothelial barriers. In one study, Jang et al. [43] developed an ENV-based AST delivery system by treating producer cells with saponins during culture, leading to the formation of nano-lipid vesicles loaded with AST. The resulting ENV-based AST formulation (EV-AST) exhibited stronger antioxidant and anti-inflammatory effects in HaCaT keratinocytes and RAW264.7 macrophages compared with free AST. These findings suggest that encapsulation not only enhanced AST stability but also increased its bioactivity. Collectively, these cases demonstrate that lipid-based vesicular systems—including liposomes and ENVs—can significantly improve the bioavailability of AST by leveraging their phospholipid bilayer structure. Such systems enable sustained release, controlled kinetics, and targeted delivery of AST, thereby overcoming its inherent physicochemical limitations and opening new avenues for precision medicine in AST applications.

### Nanoparticles

#### Preparation of nanoparticles loaded with AST

The preparation of nanoparticle-encapsulated AST can be classified into 3 methods based on the type of carrier materials, i.e., emulsification-solvent evaporation, nanoprecipitation, and ionic gelation. Emulsification-solvent evaporation [44] is the most common preparation method, and its main steps are as follows. First, AST and carrier materials are dissolved in an organic solvent, and the solution is slowly added to an aqueous phase containing surfactants. The mixture is homogenized at high speed or subjected to ultrasonic emulsification. Subsequently, the organic solvent is removed via rotary evaporation or magnetic stirring to form a nanoparticle suspension. Finally, centrifugation or dialysis purification is performed. The nanoprecipitation method involves procedures similar to those used in the emulsification-solvent evaporation method. The organic component is rapidly injected into an aqueous phase under stirring to spontaneously form nanoparticles. Finally, the organic solvent is removed by dialysis or ultrafiltration to obtain a concentrated nanoparticle suspension. In the ion gelation method, AST and COS are first dissolved in an acidic solution, and then, an aqueous phase containing a cross-linking agent is dripped into the COS solution under stirring. The formed nanoparticles are subjected to centrifugation or dialysis for purification.

#### Nanoparticles loaded with AST

Nanoparticles are microscopic structures with dimensions spanning from 1 to 100 nm. Owing to their size-dependent and surface-dominant characteristics, nanoparticles exhibit high specific surface area, multi-functionality, and modifiability. Therefore, nanoparticles have been extensively studied as drug delivery vehicles. For example, AST and ethylene glycol COS were combined to form ChitoAST nanoparticles [45] via electrostatic interaction. The nanoparticles showed excellent water solubility, enhancing the biological activity of AST. Furthermore, the loading efficiency of ChitoAST gradually increased with a rising AST feed weight. The increase in AST contents also slightly changed the zeta potential and reduced the size of nanoparticles. It was reported that the drug release rate was related to the drug content in the nanoparticles. In other words, the greater the drug loading within the nanoparticles, the more retarded the drug release kinetics. Hwang et al. [45] employed a lung metastasis model of melanoma cancer cells to verify the anti-cancer activity of AST nanoparticles (NPs). ChitoAST-2 nanoparticles exhibited antitumor and antimetastatic properties against melanoma cell lines, and the AST released from ChitoAST-2 nanoparticles suppressed the proliferation and invasion of cancer cells in a dose-dependent manner. To overcome the application limitations arising from the poor water solubility and highly unsaturated structure of AST, Yu et al. [46] fabricated carrier-free AST using the reverse-solvent method. The obtained AST-NPs not only enhanced the photostability and antioxidant properties of AST but also enabled its controlled release, thereby ensuring a continuous supply of AST to the body. The application of chlortetracycline-free AST-NPs increased the solubility of AST in water, enhanced its physical and chemical properties, effectively eliminated free radicals, and achieved a sustained and stable release of AST. Chang et al. [47] developed nanovesicles that secrete tumor cell membranes to encapsulate AST, thereby enhancing the therapeutic effect of cancer treatment. AST loaded on nanoparticles showed improved anti-cancer efficacy, and its inhibitory effect on cells increased with the concentration of nanovesicles formed by mixing AST with melanoma cancer cells (ANPs). Fluorescence staining experiments confirmed that ANPs had entered melanoma cells, inhibited cell growth, and caused death. Fluidity analysis proved that ANPs effectively inhibited the metastasis of cancer cells. In general, AST nanovesicles exhibit significantly enhanced growth inhibition against melanoma cells than AST alone, without obvious effects on normal cells. Liu et al. [48] encapsulated AST in a PLGA polymer coated with COS to enhance the solubility and stability of AST. The encapsulation yield, morphological features, zeta potentials, and particle dimensions of the synthesized Ax-PLGA@COS nanoparticles were characterized. The results revealed that the nanoparticles possessed good dispersibility, stability, and cell compatibility. Furthermore, Yang et al. [49] encapsulated AST with whey protein and COS through molecular self-assembly and rotary evaporation techniques to generate AST/bovine serum albumin/polysaccharide nanoparticles. The presence of whey protein and COS [50] could effectively enhance the stability and bioavailability of AST. However, these nanosuspensions must be frozen for convenient and safe storage, transportation, and application, which causes particle collapse and aggregation, as well as structural changes in the proteins on the particle surface. To address these issues, sugars, amino acids, alcohols, and other substances have been used to support nanoparticles. Lecithin nano-liposomal emulsion [51] is a new type of carrier for AST fabricated via a straightforward emulsion evaporation technique. Numerous experiments demonstrated that the enhanced aqueous solubility of ASTA@Les nanosuspensions significantly elevated the biopharmaceutical availability of AST, effectively scavenged ROS, and promoted wound healing without causing cytotoxicity. In addition, whey protein isolate (WPI) itself has prebiotic properties, which can regulate the intestinal flora, promote the growth of beneficial bacteria, and repair intestinal epithelial barrier function. Moreover, AST-NPs effectively alleviated intestinal fibrosis by inhibiting the TGF-β/Smad signaling pathway in a dextran sulfate sodium-induced acute/chronic colitis model (Fig. 1D) [52]. The above examples demonstrate that nano-carrier technology has advanced the application of AST through structural innovation, leveraging the excellent performance of nanoparticles to improve the chemical stability of AST.

### Self-nanoparticulate emulsified drug delivery system containing AST

The self-nano-emulsifying drug delivery system (SNEDDS) is a novel drug delivery technology that encapsulates hydrophobic drugs in nanoscale carriers through self-emulsification, which can significantly enhance the solubility, stability, and bioavailability of drugs. To prepare SNEDDS loaded with AST, AST is first mixed with surfactant under magnetic stirring and the mixture is dissolved in an oil phase. Then, a co-surfactant is added under continuous stirring, and ultrasonic treatment is performed at room temperature in a water bath for 1 h to obtain a single-month acid salt drug system containing AST. Analysis of the stability, transdermal properties, and antioxidant activity of SNEDDS [53] loaded with AST indicated that the skin permeability of AST in SNEDDS was much higher than that in the traditional system and that SNEDDS significantly improved the stability and antioxidant activity of AST. In addition, when SNEDDS comes into contact with moisture on the skin surface, it spontaneously forms extremely small oil droplets. The surfactants on their surface disrupt the orderly arranged lipid bilayer in the stratum corneum, making it easier for the nano-oil droplets that carry AST to diffuse through. The nano-oil droplets that reach the dermis interact with the skin cell membrane through mechanisms such as fusion, adsorption, or endocytosis, thus achieving precise delivery of AST. Therefore, SNEDDS is a proficient carrier that enhances the transdermal delivery of highly lipophilic AST. This is achieved by accurately delivering AST to the dermis of the target area, thereby promoting the therapeutic antioxidant and anti-inflammatory effects of AST. This nano-drug delivery technology enhances drug efficiency, reduces drug consumption, and provides opportunities for clinical translation.

### 3D-printed dressings loaded with AST

Bioactive glass microparticles (BGMs) are made from silicate glasses with specific composition and have particle sizes ranging from micrometers to nanometers. BGMs are often used as wound dressing materials owing to their excellent biological activity, antibacterial properties, ability to promote tissue repair, and stability [54]. To prepare AST-loaded BGMs [55], an acetic acid dialdehyde solution is first mixed with a gelatin solution under stirring to form a base solution. Then, AST and borate BGMs are gradually added to the base solution to obtain a pre-water gel solution for 3-dimensional (3D) printing. The printed structures might need further cross-linking and curing to enhance their stability and mechanical properties. Composite hydrogel structures formed by 3D printing are generally rigid, with a slow in vitro degradation rate, which enables stable and continuous delivery of AST. Moreover, during the 3D printing process, the positions and concentrations of AST and borate BGMs in the hydrogels can be precisely controlled according to the specific requirements. Studies found that an AST-BGM composite hydrogel not only stably delivered AST and sustained its in vitro release rate but also effectively accelerated wound healing, thereby expanding the application scope of AST. Overall, the AST-BGM obtained through 3D printing technology effectively controlled the release of AST and provided long-term protection. In addition, antibacterial borate glass can act synergistically with the anti-inflammatory AST, showing promising prospects for wound healing.

### Microcapsules loaded with AST

Microcapsules are small capsules that enclose solid, liquid, or gaseous entities made of natural or synthetic polymer materials, with the aim of establishing a physical barrier between the core substance and its environment. Emulsification-spray drying is the main method for preparing AST microcapsules. First, AST is dissolved in an appropriate oil phase, and then, an emulsifier is added to form a stable emulsion. The emulsion is sprayed onto a drying tower using a spray drying device. Under the action of hot air, the water evaporates rapidly, leaving microcapsules containing AST. AST water dispersions were prepared with PLA as the wall material. Increasing PLA concentrations on microcapsules [56] enhanced the size, encapsulation efficiency, and zeta potential of these microcapsules, while their water content, fluidity, and bulk density decreased. In addition, cell culture experiments showed that AST microcapsules based on PLA were nontoxic and had good antioxidant activity. The microcapsules have enhanced physical stability, bioavailability, and functionality, and can be used as anti-aging skin care products to reduce the damage of ultraviolet rays to the skin and delay skin aging. Calcium alginate beads, as microcapsule carriers of biopolymers, have superior mechanical strength, protective performance, and biocompatibility, and are suitable for the delivery of AST. The specific method for encapsulating AST in calcium alginate beads includes the following steps: AST is first dispersed in sodium alginate solution, which is then dripped into calcium chloride solution to form calcium alginate spherical particles. The obtained beads are separated from the solution using centrifugation and then dried. AST is encapsulated in calcium alginate microspheres by extrusion technology, effectively enhancing the stability of AST [57]. Calcium ions act as cross-linking agents for alginate chains, forming a matrix layer to prevent oxygen exposure, and cooperate with AST to improve its stability. Studies have shown that calcium alginate has anti-inflammatory effects and can work synergistically with AST to achieve better therapeutic effects. The effective protection of AST via microencapsulation in the acidic environment of gastric acid ensures its effective arrival in the intestine for absorption. Moreover, calcium alginate beads contract in an acidic environment, which effectively reduces the release of AST and thus improves its bioavailability and achieves targeted release in the intestine. Therefore, microencapsulation technology has greatly expanded the application range of AST. The technology has transformed AST from an unstable natural pigment into a stable and efficient functional component that can be used in applications such as medical care, personal care, and tissue engineering.

### AST encapsulated in polymerized micelles

Polymer micelles are thermodynamically stable colloidal dispersions formed by the spontaneous self-assembly of amphiphilic di-block copolymers in an aqueous medium. Their structures typically exhibit core–shell characteristics. The hydrophobic part forms the core for encapsulating drugs or other active substances, while the hydrophilic part constitutes the shell, enabling the micelles to remain stable in aqueous environments. To address the poor water solubility of AST, researchers discovered that hydrophobic methoxypolyethylene glycol-polycaprolactone (mPEG-PCL) could encapsulate AST to form AST-loaded mPEG-PCL micelles [58]. The main preparation method was as follows. First, mPEG-PCL is dissolved in an appropriate amount of organic solvent to form a uniform and transparent polymer solution. AST is then added to the polymer solution, followed by slowly adding water under continuous stirring. Finally, the organic solvent in the system is removed via rotary evaporation. Experimental results showed that AST encapsulated in polymer micelles reduced ROS levels in the culture system, thereby promoting the proliferation of mesenchymal stem cells (MSCs). At the optimal AST loading concentration, the micelles promoted 3-line differentiation of mesodermal cells into osteoblasts, chondrocytes, and adipocytes. In summary, using polymer micelles to encapsulate AST shows great advantages in terms of drug-loading capacity, targeting ability, and stability, providing a promising option for AST delivery.

### β-Cyclodextrin-encapsulated AST

β-Cyclodextrins (β-CDs) are generated by the action of cyclodextrin glucanotransferase, an enzyme secreted by *Bacillus* spp*.*, on linear chain amylose [59]. They usually contain 6 or 12 d-phyllan glucose units and belong to the cyclic polysaccharide family, commonly used to form inclusion complexes. To prepare AST-containing β-CD complexes, β-CD is first dissolved in an appropriate amount of water and heated under stirring to form a saturated aqueous solution. Then, AST is added to the saturated β-CD solution while stirring. Within a certain temperature range, AST reacts with β-CD to form inclusion complexes. After the completed reaction, the solution is slowly cooled to precipitate inclusion complex crystals. Finally, the crystals are filtered and dried. The obtained AST-containing β-CDs have a much higher solubility than free AST in an acidic environment. Moreover, AST-containing β-CDs have enhanced antioxidant activity, thermal stability, and water solubility. Furthermore, Xue et al. [60] prepared an AST nano-delivery system using 2-pyridyl-β-cyclodextrin and Soluplus through single-needle electrospray technology. Pharmacokinetic results indicated that this system significantly enhanced the bioavailability of AST and enabled its sustained release.

## Biomedical Applications

### Tissue engineering

#### Epidermis

AST is applied to skin tissues primarily for anti-aging and wound healing purposes [61]. The main reason for skin aging is damage to the structures and functional proteins of the ECM, including the production of active intermediates, apoptosis, and pro-inflammatory responses. In living cells, there is a balance between the activity of the antioxidant system and the generation of ROS and reactive nitrogen species (RNS). When this balance is disrupted, normal cellular structures and functions are damaged. When ultraviolet rays penetrate the skin, they cause an oxidative stress response that damages DNA, proteins, and lipids. Errors in DNA repair can lead to mutations, degradation of collagen, wrinkles, erythema, or skin cancer. Honda et al. [62] proved that AST supplementation significantly inhibited skin damage caused by ultraviolet rays. AST also effectively improved skin elasticity, texture, and moisture contents, thus reducing wrinkles and visible signs of aging [63]. Furthermore, researchers functionalized AST nano-emulsions with carboxymethyl COS, which improved the chemical stability and skin permeability of AST, thus promoting wound healing [64]. The repair process is composed of 3 interconnected phases: Phagocytosis, cell proliferation, and tissue remodeling. In vivo experiments on mice showed that, in the AST-administered group, the expression of the oxidative stress biomarker iNOS was substantially decreased. This likely inhibited the activation of the NF-κB pathway, leading to a decrease in the production of inflammatory genes and cytokines. In addition, AST might suppress the expression of adhesion molecules, thereby inhibiting inflammatory cell infiltration. In the proliferative remodeling stage, AST promoted basic fibroblast growth factor (bFGF) expression, which was crucial for the development of granulation tissue, epithelialization, matrix formation, and bFGF-related remodeling, thereby accelerating wound closure. Moreover, AST expedited wound closure in the proliferative and remodeling phases by strengthening the activity of contractile fibroblasts. Researchers prepared oil–water nano-emulsions containing AST with uniform stability using spontaneous and ultrasonic emulsification methods. The anti-cancer, wound healing, and antibacterial effects exhibited by these nano-emulsions [65,66] have made them promising alternative biomaterials to treat chronic wounds in diabetic patients.

#### Vascular tissue

Vascular endothelial cells line the interior cavities of all vascular channels and perform diverse functions in preserving homeostasis of the cardiovascular system [67]. Endothelium impairment triggers many pathologies and is correlated with redox imbalance and angiitis. Researchers used Raman spectroscopy and fluorescence microscopy to study the uptake of free and liposomal AST into endothelial cells [67]. Studies have shown that liposomes enhance the uptake of AST. The anti-inflammatory effect of liposomal AST successfully reduced lipid unsaturation in endothelial cells, decreased the number of lipid droplets, and inhibited high expression of ICAM-1, a common indicator of endothelium-related inflammation.

#### Muscle tissue

Sarcopenia is a progressive and systemic disorder marked by decreased mass of skeletal muscles, diminished muscular strength, and impaired muscle function. Most molecular and cellular changes that lead to muscle degeneration are regulated by ROS-induced stress [68]. AST can serve as a potential adjuvant therapy for sarcopenia owing to its antioxidant properties. Studies have shown that the mitochondrial electron transport chain is the primary site of ROS generation in skeletal muscles. Therefore, mitochondrial DNA is susceptible to oxidative injury owing to the excessive generation of ROS, thereby impacting the homeostasis of mitochondria and their physiological function. Abundant PGC-1α serves as an index of optimized oxidative metabolism and mitochondrial physiological activity. Studies found that AST accelerated lipid utilization in skeletal muscle by increasing PGC-1α levels and reducing the pH value in muscles during aerobic exercise. In addition, AST enhanced energy production and effectively extended exercise duration. These studies also demonstrate that dietary AST supplementation can prevent nuclear muscle cell apoptosis in the soleus muscle, thereby helping to preserve muscle mass.

#### Cartilage tissue

Osteoarthritis is a joint disease characterized by the destruction of subchondral bone remodeling [69], leading to microstructure changes, degeneration, and damage of articular cartilage, as well as reactive hyperplasia of the articular margin and subchondral bone. Inflammatory cytokines, such as TNF-α or IL-1β, initiate a series of different signal transduction pathways. This stimulation induces cartilage damage and disrupts the dynamic balance of the cartilage matrix by generating chondrolysase, MMPs, and a disintegrin and metalloproteinase with thromboreactive protein motifs. These enzymes drive chondrocytes into a catabolic state, thereby leading to the onset of osteoarthritis. Tripathi and Jena [70] found that AST inhibited the degradation of cartilage and the apoptosis of chondrocytes caused by oxidative stress through up-regulating the expression of matrix-degrading enzymes. Moreover, the accumulation of excessive iron promoted the production of ROS, thereby leading to a series of orthopedic diseases, including osteoarthritis. Using 10 μM AST could protect chondrocytes and delay the development of osteoarthritis by reducing the proliferation of iron cells in chondrocytes.

**Fig. 2.**
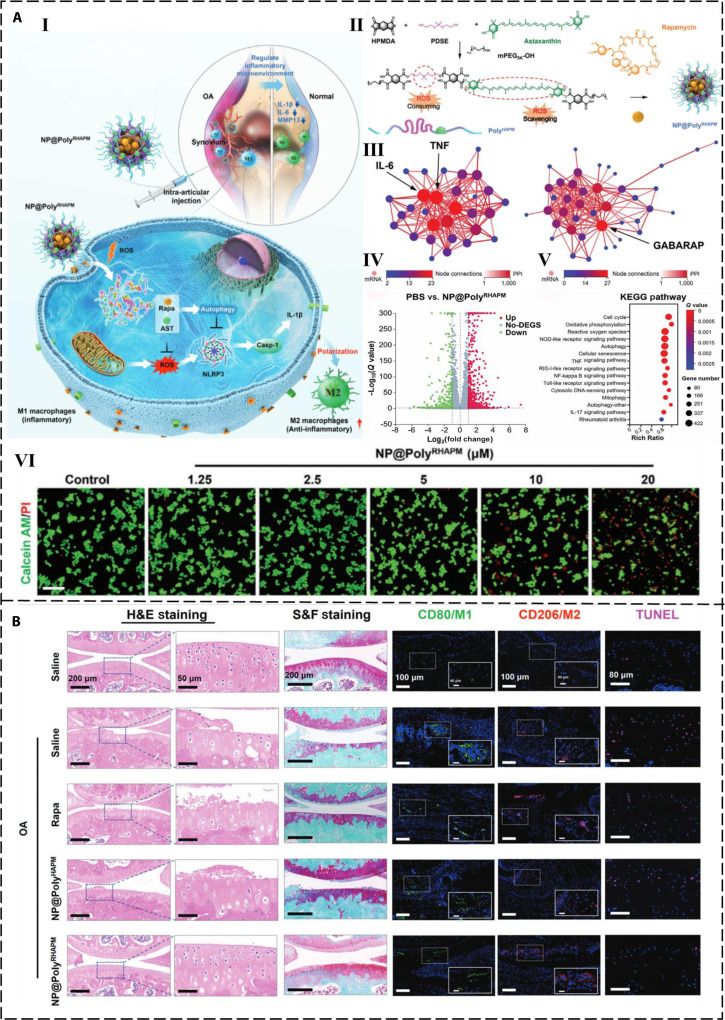
Mechanism of action, preparation method, and effects of NP@PolyRHAPM. (A) (I) Schematic illustration of the effect of NP@PolyRHAPM on polarizing macrophages in knee osteoarthritis. (II) Investigation of the anti-inflammatory mechanism of NP@PolyRHAPM. (III) Protein–protein interaction network analysis of differentially expressed core genes involved in the positive regulation of interleukin-1β (IL-1β) production and autophagy. (IV) Scatterplot showing differentially expressed genes after NP@PolyRHAPM treatment. (V) Analysis of the enriched bubble chart of Kyoto Encyclopedia of Genes and Genomes (KEGG) pathways. (VI) Live/dead staining images show the effect of drug concentration on the activity of macrophages. (B) Hematoxylin and eosin staining and Safranin O and Fast Green staining (S&F staining) results demonstrating that NP@PolyRHAPM could alleviate pathological damage in an osteoarthritis model [71].

Li et al. [71] designed a ROS-responsive nanoparticle (NP@PolyRHAPM). The biodegradable PolyHAPM was synthesized by condensation polymerization of AST, 2,2′-(propane-2,2-diylbis(sulfanediyl)) bis(ethan-1-ol) (PDSE), and 1,2,4,5-cyclohexanetetracarboxylic dianhydride (HPMDA), followed by sealing the ends with mPEG5k-OH. Finally, NP@PolyRHAPM was obtained by encapsulating rapamycin with PolyHAPM. The effect of different doses of NP@PolyRHAPM on macrophages was examined by live/dead staining, confirming that NP@PolyRHAPM was able to attenuate the inflammatory response of macrophages. Furthermore, the anti-inflammatory mechanism of NP@PolyRHAPM was analyzed through RNA sequencing. NP@PolyRHAPM exerted its anti-inflammatory effects by directly eliminating excessive ROS in M1 macrophages, strengthening subsequent autophagy pathways, and inhibiting the secretion of pro-inflammatory cytokines (Fig. 2A, I to VI). These effects are all beneficial for the treatment of osteoarthritis. In addition, cell morphology and tissue structural integrity were examined through hematoxylin and eosin staining, which revealed that NP@PolyRHAPM successfully slowed down the destruction of cartilage in osteoarthritis. The content of proteoglycans in cartilage tissue was also analyzed through Light Green SF Yellowish staining to understand the degree of cartilage degeneration (Fig. 2B). Overall, NP@PolyRHAPM inhibited synovial inflammation by regulating the expression of inflammatory factors, thereby protecting chondrocytes from damage.

#### Bone tissue

MSCs [72] have been extensively utilized in tissue engineering and regenerative medicine owing to their distinctive self-replication and pleiotropic properties. Research indicated [73] that high ROS levels reduced the mitotic activity and self-replication competence of MSCs. AST, as a potent antioxidant, can eliminate excessive ROS in the intercellular space, thereby providing a relatively stable internal environment for cells and promoting the proliferation of MSCs [74]. Furthermore, AST regulates intracellular signaling pathways, such as activating the bone morphogenetic protein-2 (BMP-2)/Smad signaling pathway. BMP-2 is an important inducer of osteogenesis. When this pathway is activated by AST, it can enhance the differentiation of MSCs into osteoblasts. AST also affects the wingless-related integration site/β-catenin signaling pathway, which is pivotal for the specialization and mitotic expansion of bone-forming cells. The regulation of this pathway by AST is conducive to promoting the transformation of MSCs into osteoblasts.

Tao et al. [75] have shown that palmitic acid (PA) is one of the most common saturated fatty acids in the bone marrow of osteoporotic women and may exert lipid toxicity on bone metabolism. In osteoporosis animal models, PA has been shown to accelerate bone loss and reduce bone strength. Bone repair is closely related to the activity of osteoblasts [76]. The inhibition of osteoblast differentiation and the promotion of osteoclast activity are mainly due to the generation and accumulation of excessive ROS [77]. Research confirmed that in ovariectomy animal models, AST reduced oxidative stress levels in serum, protected bone mass, and prevented a decline in bone density. However, the mechanisms and signaling pathways of AST and PA remain unclear. Osteogenic sarcoma is a cancerous neoplasm of osseous tissue that arises from connective tissue mesenchyme [78]. Owing to mutations, excessive bone resorption and abnormal activation of the wingless signaling pathway led to uncontrolled expression of oncogenes, creating conditions for tumor progression. Studies have shown that AST significantly reduced the cell proliferation of 3 canine osteosarcoma cell lines, OS 2.4, HMPOS, and D17, by up-regulating the expression of Runx2 [79], type I collagen, osteopontin, and osteocalcin in MG63 cells, and inhibited the mitotic activity of osteosarcoma cells [80]. These results indicate that AST has an anti-proliferative effect by inhibiting tumor cell proliferation and inducing chemoprophylactic apoptosis.

#### Immune system

As a complex defense network within the human body, the immune system recognizes and eliminates foreign pathogens, abnormal cells, and harmful substances produced within the body, thereby maintaining immune homeostasis. The core cause of common immune-related diseases such as sepsis, systemic lupus erythematosus, and rheumatoid arthritis is the disruption of immune homeostasis [81]. Sepsis is a systemic immune response triggered by an infection that subsequently leads to organ dysfunction and life-threatening conditions. The pathological mechanism of sepsis is that lipopolysaccharides (LPSs) released by gram-negative bacteria activate TLR4 receptors on immune cells and trigger an inflammatory factor storm. Immunostimulation and immunosuppression have become primary therapeutic strategies for sepsis [82]. Dendritic cells, as the most potent cells responsible for antigen presentation in the immunological system, play an important role in balancing immune homeostasis and controlling the deviation of downstream immune responses. Studies have shown that the dynamic changes in dendritic cells are closely linked to the immune imbalance in sepsis [83], making them potential therapeutic targets for treating sepsis. Yin et al. [84] discovered that AST inhibited LPS-induced cytokine secretion by dendritic cells, reducing their phenotypic maturation and enhancing their endocytic and migratory abilities. More importantly, AST also suppressed the immunological disorder of antigen-presenting dendritic cells caused by LPS through stimulating the HO-1/Nrf2 signaling pathways. This intervention significantly improved the survival rate of mice exposed to LPS. These results strongly support the efficacy of AST in the treatment of sepsis.

He et al. [85] also found that AST could alleviate autoimmune hepatitis (AIH) by regulating CD8^+^ T cells. AIH is a chronic liver inflammation caused by an abnormal immune system attack on liver cells. The main pathogenesis of AIH is immune system imbalance, and T cells play a pivotal role in the advancement of AIH [86]. Researchers constructed a concanavalin A (ConA)-induced AIH mouse model and found that AST regulated the quantity and function of CD8^+^ T cells. That study indicated that AST inhibited pro-inflammatory responses and alleviated liver damage in a mouse model, providing a new therapeutic strategy for the treatment of immune-related diseases. Overall, AST has a multidirectional regulatory effect on the immune system through antioxidative, anti-inflammatory, and immunomodulatory mechanisms. It can enhance immune defense capabilities and inhibit excessive immune responses. Therefore, AST shows great potential in preventing infection and managing chronic inflammation, serving as an adjunctive treatment for autoimmune diseases.

### Drug delivery

#### Brain and nervous system

Neurological disorders [87,88] refer to various diseases related to the nervous system, including the central nervous system, peripheral nervous system, and lesions in the neuromuscular junction, muscles, and other parts. Acute injury and chronic neurodegenerative disorders are common causes of mortality and morbidity in humans. The etiopathogenesis of these conditions is linked to oxidative stress, inflammatory responses, and apoptosis.

The main cause of Alzheimer’s disease is the accumulation of amyloid-β peptide, which leads to significant oxidative damage in the patient’s brain [89]. In addition, mitochondrial abnormalities and hyperphosphorylated tau can also trigger oxidative and inflammatory responses, causing Alzheimer’s disease [90]. Intake of AST can reduce the oxidation index of the brain and help maintain the normal functions of nerve cells, thereby alleviating the symptoms of Alzheimer’s disease [91]. The immunomodulatory anti-inflammatory attributes of AST can impede the hyperactivation of microglial cells, reduce the release of inflammatory factors, and delay the progression of Alzheimer’s disease. In addition, AST regulates the signaling pathways in the brain, promotes the production of neurotrophic factors, and thereby repairs damaged neurons. For instance, Santonocito et al. [92] loaded AST onto stealth lipid nanoparticles (AST-SSLNs) using solvent diffusion technology. These nanoparticles exhibited enhanced antioxidant capacity and stability compared to free AST, making them well suited for systemic AST administration for Alzheimer’s disease treatment.

**Fig. 3.**
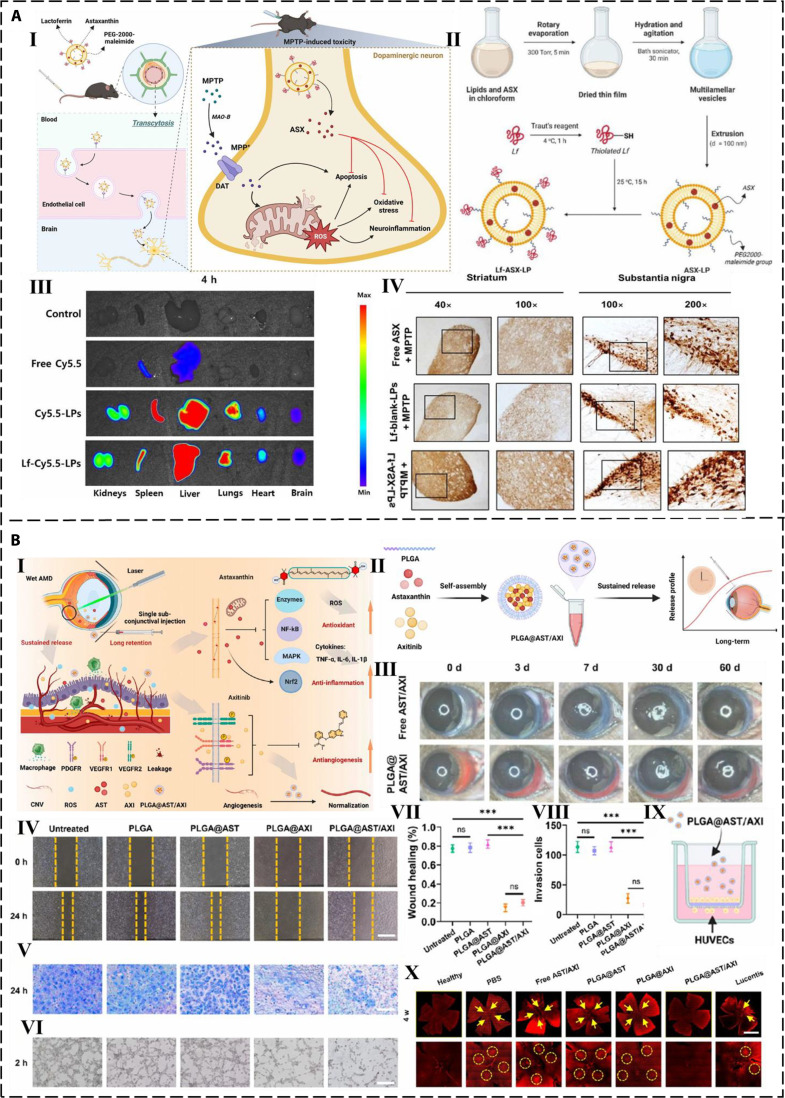
Mechanism of action, preparation method, and therapeutic effect of LF-ASX-LPs and PLGA@AST/AXI. (A) (I) Scheme illustrating the mechanism of action of LF-ASX-LPs in the treatment of Parkinson’s disease. (II) Scheme of the preparation process of LF-ASX-LPs. (III) Accumulation of Cy5.5 fluorescent probe in the brain and other organs 4 and 24 h after injection. (IV) Expression of TH^+^ fibers in the striatum and quantification of TH^+^ dopaminergic fibers in the striatum [95]. (B) (I) Scheme illustrating the mechanism of action of PLGA@AST/AXI in the treatment of AMD. (II) Scheme of the preparation of PLGA@AST/AXI and its long-term sustained drug release characteristics. (III) Images of the eye in mice after injection with free AST/AXI or PLGA@AST/AXI at different time points. (IV and VII) Scratch assay images and quantitative analysis demonstrating that PLGA@AST/AXI promotes cell migration and accelerates tissue repair. (V) Transwell invasion assay showing invasive ability. (VI, VII, VIII and IX) The tube formation assay, migration assay, Transwell invasion assay images, and quantitative analysis of HUVECs across all experimental groups collectively demonstrated that the PLGA@AST/axis significantly inhibits angiogenesis. (X) IB4 staining of retinas showing that PLGA@AST/AXI induces regression of neovascularization [102].

Parkinsonism predominantly results from the neurodegeneration and apoptosis of dopaminergic neurons in the substantia nigra pars compacta of the mesencephalon [93]. Parkinson’s disease is also related to genetic factors, environmental factors, and aging of the nervous system. AST can effectively eliminate ROS and alleviate the damage caused by oxidative stress to dopaminergic neurons in the substantia nigra of the mesencephalon, thereby slowing the progression of Parkinson’s disease. By suppressing the inflammatory cascade in the brain and reducing the production of inflammatory factors, AST can reduce inflammatory harm to neurons, thereby alleviating the symptoms of Parkinson’s patients. Radonin is an organic pesticide with the same action mode on neurons as Parkinson’s disease, both of which induce neurotoxicity by inhibiting mitochondrial complex I and increasing oxidative burden, thereby affecting dopaminergic neurons. Anguchamy and Muthuvel [94] assessed the efficacy of AST in mitigating radon-induced toxicity in SK-N-SN human neuroblastoma cell lines. They found that AST alleviated radonin-induced cytotoxicity, mitochondrial dysfunction, and oxidative stress in a dose-dependent manner. Therefore, AST can serve as a supportive treatment for Parkinson’s disease and other degenerative diseases. Furthermore, Nguyen et al. [95] designed lactoferrin-conjugated AST liposomes (Lf-ASX-LPs) for neuroprotection, aiming to slow down the progression of Parkinson’s disease. In that study, ASX-loaded liposomes (ASX-LPs) were prepared using the thin-film hydration method and subsequently conjugated with lactoferrin via a thiol-maleimide coupling reaction. The researchers also characterized the liposomes and confirmed their high encapsulation efficiency and excellent physical properties (Fig. 3A, I and II). Furthermore, 24-h in vivo imaging in mice revealed the distribution of the Cy5.5 fluorescent probe in various organs, demonstrating that these liposomes had high penetration rate. The protective effect of Lf-ASX-LPs on dopaminergic neurons was evaluated under neurotoxicity induced by 1-methyl-4-phenyl-1,2,3,6-tetrahydropyridine (MPTP). The results showed that Lf-ASX-LPs provided significantly greater neuroprotection than free ASX (Fig. 3A, III and IV). Overall, Lf-ASX-LPs have proven effective in delaying Parkinson’s disease by alleviating neuroinflammation, oxidative stress, and mitochondrial dysfunction.

Cerebral ischemia refers to insufficient blood supply to the brain due to disrupted blood circulation. The causes of cerebral ischemia include narrowed blood vessels, the formation of blood clots within the blood vessels, or vasospasm. During cerebral ischemia, a large amount of free radicals is produced, thereby causing damage to nerve cells [96]. AST provides electrons that can react with free radicals, thereby facilitating their transformation into more stable species, which, to a certain extent, protects nerve cells. From the perspective of anti-apoptosis, AST can reduce the cell death rate related to cerebral infarction by up-regulating Bcl-2 and inhibiting Bax. However, AST can only be used as an auxiliary treatment and cannot replace conventional cerebral ischemia treatment plans.

Subarachnoid hemorrhage refers to the rupture of blood vessels at the bottom or surface of the brain, causing bleeding directly into the subarachnoid space. As an acute cerebrovascular disease, it is characterized by a sudden onset of symptoms and rapid progression, often leading to death. Subarachnoid hemorrhage is more common in middle-aged and elderly individuals, with symptoms such as headaches, vomiting, and weakness [97]. However, the cause of subarachnoid hemorrhage remains unknown. AST can inhibit inflammatory factors and pro-apoptotic cytokines by modulating inflammatory and oxidative stress responses, thereby enhancing the survival rate of neurons in the arachnoid membrane. For instance, Cai et al. [98] reported that nano-sized ultrasound-triggered release of nanoparticle-encapsulated AST exhibited good biocompatibility and effectively enhanced the bioavailability of AST, enabling its accurate release in neuronal cells.

Amyotrophic lateral sclerosis (ALS) [99], also known as Lou Gehrig’s disease, is a progressive neurodegenerative disorder that predominantly impacts upper and lower motor neurons within the central nervous system, encompassing the cerebral cortex and spinal cord. In ALS patients, the upper and lower motor neurons within the cerebral cortex and spinal cord undergo progressive neurodegeneration and ultimate apoptosis, preventing the muscles from receiving instructions from the brain, which in turn leads to muscular wasting and asthenia. Although the pathogenic mechanisms underlying ALS remain unclear, the most common cause is related to a gene mutation of SOD1, which results in the accumulation of free radicals. AST can provide electrons for reactions with free radicals, inhibiting neuronal death related to the accumulation of free radicals. According to previous research, antioxidant compounds can serve as pharmacotherapeutic agents, potentially enhancing the functional status and overall quality of life for individuals with ALS. In addition, clinical dietary research suggests that the intake of carotenoids, such as AST, may improve the respiratory function in ALS patients.

#### Eye

When light passes through each eye layer, large amounts of ROS are produced. Moreover, elevated intraocular pressure, inflammatory stimulation, and other factors accelerate oxidation reactions [100]. Common carotenoids can neutralize single oxygen molecules and eliminate free radicals to prevent chain reactions. Among them, AST exhibits superior antioxidant activities, thus effectively preventing the formation of ROS and eliminating hydroxyl radicals. This effect successfully reduces the death rate of retinal ganglion cells [101] and increases the stability of ocular surface units. Overall, AST helps protect retinal cells from oxidative damage and ultraviolet radiation. Most drugs currently used to treat eye diseases are administered to the surface and front of the eyes. In vivo studies indicate that biocompatible and biodegradable nanoparticles are ideal drug carriers to deliver AST to the posterior region of the eye [22]. Moreover, they can extend the shelf life and enhance the absorption of AST.

AMD is a chronic degenerative eye disease, primarily affecting the central area of the retina known as the macula. Chang et al. [102] have developed a new multifunctional nano-drug delivery system specifically targeting AMD. To synthesize multifunctional nanoparticles, AST was co-loaded with an oral small molecule targeted anti-cancer drug (AXI) to the center of PLGA. At different time points, PLGA @AST/AXI was injected into the eyes of mice with free AST/AXI as a control. It was found that PLGA@AST/AXI accumulated in the conjunctiva and continuously released the drug for 2 months (Fig. 3B, I to III). Furthermore, the in vitro therapeutic effect of PLGA@AXI/AST was investigated through wound scratch assays and Transwell invasion assays for different groups of human umbilical vein endothelial cells. These assays are commonly employed to assess cell viability and antioxidant capacity. The results confirmed that PLGA@AST/AXI nanoparticles retained strong antioxidant capacity in vitro and demonstrated a therapeutic effect in treating laser-induced choroidal neovascularization in mice by inhibiting vascular proliferation. Histological examination revealed no obvious pathological abnormalities in the eyes and major organs of the mice (Fig. 3B, IV to X), proving that PLGA@AST/AXI is a safe reagent.

#### Lungs

Asthma is a common chronic inflammatory airway disease, mainly characterized by the infiltration of eosinophils, mast cells, T lymphocytes, macrophages, histamines, leukotrienes, and cytokines (such as IL-4, IL-5, and IL-13), leading to vasodilatation, increased mucus secretion, and airway hyperresponsiveness. Its pathogenesis is associated with increased inflammation in the lungs. Cheng and Eroglu [103] reported that AST reduced oxidative markers and oxidative stress in the lung tissue of asthmatic mice to indirectly inhibit the triggering of NF-κB and other pro-inflammatory signaling pathways, thereby reducing inflammation. Furthermore, AST reduced the release of cytokines and eosinophil infiltration and suppressed the production of prostaglandin E_2_ (PGE_2_) and nitric oxide (NO) by inhibiting the expression of COX-2 and iNOS. In addition, AST reduced immunoglobin E levels and inhibited mast cell degranulation in ovalbumin-induced asthmatic mice.

Chronic obstructive pulmonary disease (COPD) is characterized by chronic bronchitis or emphysema with airflow obstruction. The main causes of COPD are smoking, air pollution, and other factors, which lead to enhanced oxidative stress in the lungs, excessive ROS, and destruction of alveolar and airway tissues. In vivo studies [104] demonstrated that AST played an important role in the treatment of COPD as it directly scavenged ROS, alleviated oxidative damage, and enhanced the endogenous antioxidant system. In addition, AST can reduce the secretion of neutrophil chemokines such as CXCL1 and CXCL8, thereby hindering the aggregation of inflammatory cells. Inhibiting mitochondrial-dependent apoptotic pathways (such as a decrease in the Bcl-2/Bax ratio) can increase the survival rate of alveolar cells.

Acute lung injury (ALI) is a diffuse pulmonary inflammatory syndrome triggered by a variety of factors, including pulmonary edema, hypoxemia, and reduced lung compliance. Several studies showed that AST reduced lung congestion, inflammatory cell infiltration, alveolar wall thickening, and interstitial edema, thereby inhibiting the occurrence of ALI [15,105]. AST also ameliorated pulmonary fibrosis to a certain extent. The core mechanism underlying pulmonary fibrosis is excessive ROS levels, leading to DNA damage, lipid peroxidation, and protein oxidation, which in turn promote fibroblast activation and collagen deposition. Several studies suggested that AST could neutralize superoxide anions and hydroxyl radicals, thereby inhibiting oxidative stress damage in alveolar epithelial cells. Animal experiments have shown that AST reduces the content of hydroxyproline, a marker of collagen deposition in the lung tissue of bleomycin (BLM)-treated models, demonstrating that AST can decrease the synthesis of collagen and fibronectin [106]. Cao et al. [107] encapsulated AST and siTGF-β1 into ionizable liposome nanoparticles to form ionizable liposome nanoparticles (ASNPs) using microfluidic devices. This nano-delivery system effectively treated idiopathic pulmonary fibrosis (IPF) by significantly inhibiting activation of pulmonary fibroblasts and oxidative stress-induced damage to type 2 alveolar epithelial cells.

Wang et al. [108] developed pulmonary surfactant (PS) nano-biologically inspired liposomes, which were loaded with AST and an anti-fibrosis drug pirfenidone (PFD). The resulting PS nano-biomimetic liposomes (PSBs) were inhaled by patients in an aerosol form, thereby inhibiting the progression of IPF. To further study the anti-fibrotic effect of PSBs@AST/PFD on mice after silica damage, α-smooth muscle actin (α-SMA) protein levels were measured to test the fibrotic response. The results showed that α-SMA in the PSBs@AST/PFD group decreased. Masson staining of the lungs showed that collagen deposition was reduced in the PFD group, whereas it remained significantly higher than that in the normal group. In contrast, collagen deposition in the PSBs/PFD@AST group was comparable to that in BLM groups with no significant difference from the normal group. This proved that the anti-fibrotic effect of PSBs@AST/PFD was stronger than that of BLM. Furthermore, the anti-fibrotic mechanism of PSBs@AST/PFD was explored. Transmission electron microscopy images of ACE2 in lung tissues after treatment showed that the morphology of the lamellar bodies in the PSBs@AST/PFD group was close to normal. Overall, PSBs@AST/PFD effectively inhibited the expression of α-SMA and reduced the levels of coarse fibrosis factors, thereby controlling the progression of IPF.

#### Heart

Cardiac injury is the initiating factor of fibrosis, which further impairs cardiac function, leading to a vicious cycle. Shatoor and Al Humayed [109] demonstrated the anti-oxidative, anti-inflammatory, and anti-fibrotic effects of AST on high-fat diet-induced heart injury by activating SIRT1 signaling. Mitochondria are important organelles that account for 30% to 40% of the myocardial cell volume and are the main source of adenosine triphosphate (ATP). Abnormal opening of the mitochondrial permeability transition pore (mPTP), a highly conductive channel in the inner mitochondrial membrane, is one of the core pathological mechanisms of heart failure. Its activation leads to mitochondrial membrane potential collapse, disrupted energy metabolism, and eventually cell death. Mitochondrial dysfunction can cause oxidative stress, which is a contributing factor in the development of cardiovascular diseases [110]. Krestinina et al. [111] proved that AST directly penetrated mitochondria, thereby inhibiting the abnormal opening of mPTP, preventing cell apoptosis, mitigating the release of inflammatory factors, reducing myocardial inflammatory infiltration, and providing protection against oxidative damage to cardiac tissues.

In addition, alcoholic cardiomyopathy (ACM) is a disease characterized by myocardial cell degeneration, necrosis, and fibrosis, resulting from long-term excessive alcohol consumption. Wang et al. [112] constructed an animal model and demonstrated that AST hindered the progression of ACM by inhibiting endoplasmic reticulum stress-mediated and ethanol-induced apoptosis of cardiac cells. In prediabetes, hyperglycemia-induced ROS induce oxidative stress, which in turn leads to cardiovascular diseases due to thrombosis, arteriosclerosis, vascular damage, and platelet aggregation. Ciaraldi et al. [113] found that after AST treatment, low-density lipoprotein and total cholesterol levels were reduced in prediabetic patients, and the levels of cardiovascular risk markers (fibrinogen and L-selectin) were also decreased. Clinical evidence [114] also confirmed the therapeutic effects of AST in the prevention and treatment of cardiovascular diseases. At the molecular level, AST can effectively eliminate free radicals, reduce oxidative stress, and improve the bioavailability of NO and the activity of antioxidant enzymes, thereby improving vascular endothelial function. In terms of anti-inflammation, AST inhibits the release of pro-inflammatory factors and promotes high-density lipoprotein-mediated reverse cholesterol transport by regulating the key NF-κB and MAPK signaling pathways, thereby reducing foam cell formation and atherosclerotic plaque deposition. Hypertension is one of the main factors that lead to cardiovascular diseases. Previous animal experiments have shown that AST has a protective effect against deoxycorticosterone acetate salt-induced hypertension by inhibiting DNA fragmentation and cell apoptosis [115]. It is worth noting that existing preclinical and clinical studies have confirmed that AST has an excellent safety profile, which makes it not only a primary prevention strategy but also a potential adjuvant treatment for cardiovascular diseases. Based on current evidence, AST has broad application prospects in cardiovascular health maintenance and disease intervention.

#### Breast

Breast carcinoma is the most prevalent malignant neoplasm among women globally. Its incidence is positively correlated with the human development index, and the survival rate varies significantly among countries, reflecting a gap in medical resources, diagnosis, and treatment levels. Recent studies have shown that AST has multi-target regulatory potential in the process of treating breast cancer and become a research hotspot owing to its ability to inhibit proliferation, induce apoptosis, and regulate the tumor microenvironment. The main research directions of using AST for breast cancer treatment include exploring anticancer mechanisms, developing combination therapies, and applying nano-delivery systems.

In terms of the anticancer mechanism of AST, in vitro studies showed that AST selectively impeded the proliferation of breast cancer cells in a dose-dependent manner [116], with no obvious toxicity to nontumorigenic cells. Its action mechanism involves the activation of apoptosis pathways, inhibition of angiogenesis, regulation of lipid metabolism, and modulation of the inflammatory response [117]. AST exhibited varying effects across different molecular subtypes of breast cancer cells. For example, AST had a particularly significant anti-migration effect on triple-negative breast cancer MDA-MB-231 cells due to its high metastatic characteristics [118]. In HER2-positive SKBR3 cells [119], AST down-regulated the expression of HER2 and oncogenic proteins. In estrogen receptor-positive (ER-positive) MCF-7 cells, ASX suppressed the malignant phenotype by reducing the levels of paxillin and mutant p53. Notably, AST, in combination with β-carotene and lutein, synergistically enhanced MCF-7 cytotoxicity, induced G0/G1 phase arrest, and activated apoptotic pathways without causing significant damage to normal mammary epithelial cells. In vivo animal experiments confirmed that dietary AST supplementation significantly reduced the incidence of breast tumors induced by carcinogens in rats, and the mechanism was related to the regulation of adipokines and the maintenance of tissue integrity. AST produced synergistic effects when it was combined with chemotherapy drugs or bioactive substances. For example, AST combined with doxorubicin enhanced apoptosis in MCF-7 cells by regulating the redox balance while reducing oxidative stress in normal cells. Combination treatment with carbendazim synergistically induced G2/M phase arrest and decreased ROS levels. However, the interaction between AST and chemotherapeutic drugs is cell line-specific, and some combinations may impair the efficacy of chemotherapy, highlighting the importance of careful screening when designing therapeutic regimens.

The innovative application of nano-delivery systems provides an effective solution to addressing the poor aqueous solubility and low bioavailability of AST. AST-loaded gold nanoparticles (AST-AuNPs) [120] significantly enhanced cellular uptake and cytotoxicity compared to free AST. Heparin-AST/adriamycin nanoparticles (LA/DOX NPs) inhibited the formation of neutrophil extracellular traps and effectively reduced liver metastasis in breast cancer [117]. COS oligosaccharide/alginate nanoparticles loaded with AST (AST-COANPs) could improve the anti-inflammatory activity and cytotoxicity of AST, opening new avenues for the development of AST-containing functional foods and drugs.

In summary, AST shows multidimensional regulatory potential in treating breast cancer. However, the clinical translation of AST requires additional efforts to optimize combination therapy, improve delivery efficiency, and develop personalized treatment strategies. Future work should analyze the molecular network of AST and develop precision treatments based on the molecular classification of tumors.

#### Stomach

Gastric ulcers are a common digestive system disease mainly caused by long-term *Helicobacter pylori* infection [121] and gastric mucosal damage due to the overuse of nonsteroidal anti-inflammatory drugs. In response, neutrophils accumulate in inflammatory areas and produce ROS and RNS. Therefore, the severity of gastric ulcers is related to oxidative stress. Lee et al. [122] reported that AST effectively reduced ROS in gastric epithelial cells by activating PPAR-γ and inducing the expression of catalase, thereby inhibiting ROS-mediated mucosal damage. Therefore, AST exhibited an inhibitory effect on the development of gastric ulcers. In addition, gastric cancer tumorigenesis is strongly related to oxidative stress and inflammation-induced damage to the cell membrane, DNA, and proteins. Notably, elevated ROS levels not only induce apoptosis by regulating p38 MAPK and activating downstream signaling targets, thus promoting gastric cancer development, but also activate redox-sensitive transcription factors such as NF-κB. This, in turn, enhances the expression of inflammatory genes and adhesion molecules, thereby facilitating the invasion of gastric cancer cells.

#### Liver region

AST exhibits a protective effect against liver fibrosis. Liver fibrosis is a liver lesion caused by long-term damage to the liver due to various pathogenic factors, such as viruses, alcohol, drugs, and autoimmune factors [123]. It disrupts the physiological architecture and hepatobiliary function of the liver, progressively leading to chronic hepatic disorders and ultimately hepatic cirrhosis. Studies [124] have shown that AST inhibits the activation of hepatic stellate cells and the synthesis of ECM by down-regulating the expression of NF-κB and TGF-β1, as well as by preserving the equilibrium between MMP-2 and tissue inhibitor of metalloproteinase 1. The combined use of liposomes and AST effectively inhibited liver fibrosis. Moreover, in the 1990s, researchers discovered that AST could prevent oncogenesis and inhibit cell proliferation and metastasis. Gradelet et al. [125] demonstrated in an aflatoxin B1-induced hepatocarcinogenesis murine model that AST impeded the initiation and progression of hepatocellular carcinoma by inhibiting the covalent binding of aflatoxin B1 to hepatic DNA and plasma proteins. Song et al.’s [126,127] research in 2010 demonstrated that in a rat model of early liver cancer induced by cyclophosphamide, AST effectively reduced the number and size of liver cancer lesions by regulating the Nrf2/ARE pathway. Moreover, Shao et al. [128] elucidated that AST primarily inhibited cellular proliferation and induced apoptosis both in vivo and in vitro by impeding cell cycle progression at the G2/M transition phase. Ohno et al. studied the regulation of fatty acid synthase by AST using a mouse model. They found that AST increased the serum adiponectin concentration, reduced the levels of reactive oxygen metabolites, and enhanced the potential of biological antioxidants, thereby highlighting the effects of AST in treating liver tumors in obese individuals [129].

AST also prevented hepatic steatosis ischemia–reperfusion injury [130]. The core mechanisms of ischemia–reperfusion injury involve 5 aspects: ROS burst, intracellular calcium overload, amplification of the inflammatory response, mitochondrial dysfunction, and microcirculation disorders. Researchers constructed a high-fat model in mice by providing them with a methionine- and choline-deficient high-fat diet. In this experimental model, AST reduced the levels of serum alanine aminotransferase and aspartate aminotransferase, and decreased the number of terminal deoxynucleotide transferase deoxyuridine triphosphate gap terminal marker-positive cells. AST further reduced the number of macrophage-specific cell surface marker F4/80-positive cells and the level of pro-inflammatory cytokine mRNA. In addition, AST increased the phosphorylation of HO-1 and enhanced the expression of HIF-1α, Akt, and mTOR. In conclusion, AST pretreatment has a protective effect against fatty liver ischemia–reperfusion injury and is effective in managing fatty liver during liver transplantation.

**Fig. 4.**
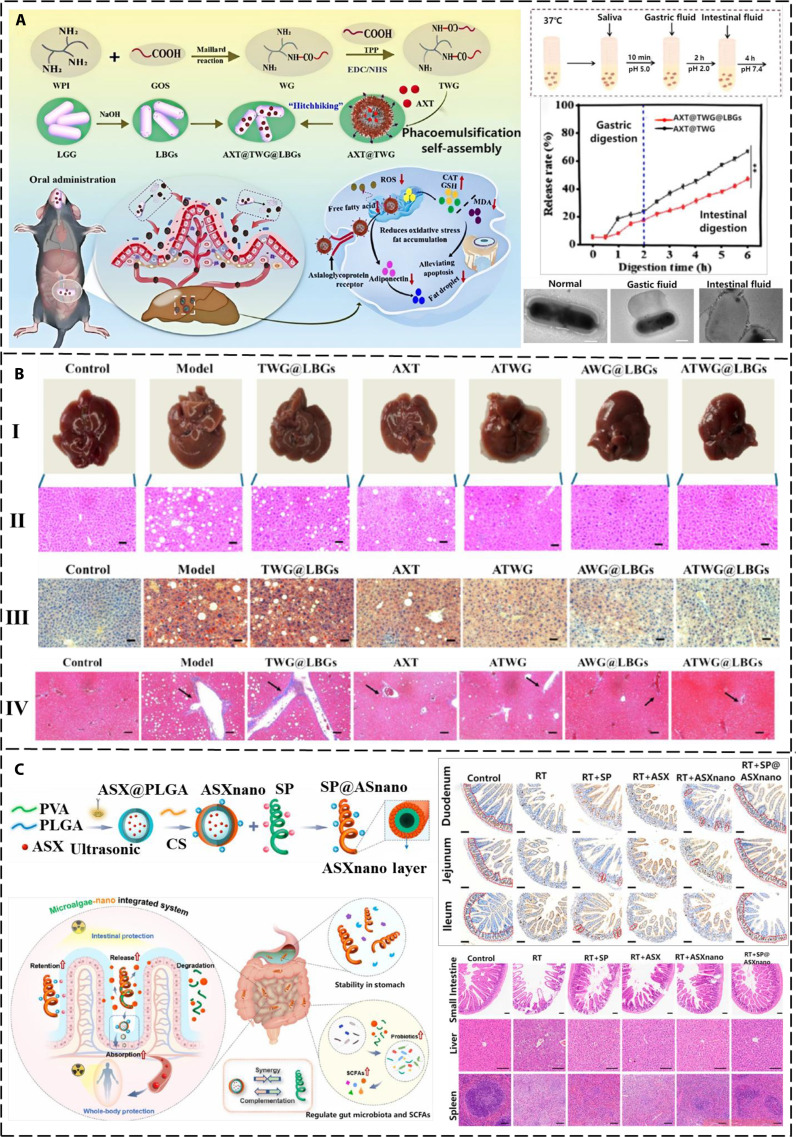
Preparation, characterization, and advantages of dual-target AXT@TWG@LBG and microalgae nanointegrated system (SP@ASXnano). (A) Preparation, simulated digestion diagram, digestion curve, and transmission electron microscope images of LBG digestion at different times. (B) Efficacy of AXT@TWG@LBG and AXT@TWG on non-alcoholic steatohepatitis. (I) Morphology of mouse liver. (II) Hematoxylin and eosin staining pathological section. (III) Oil red O staining of the liver tissue section. (IV) Masson staining of mouse liver sections showing that AXT@TWG@LBG significantly reduces the accumulation of liver fat and alleviates the progression of fibrosis [147]. (C) SP@ASXnano assembly methodology with mechanistic insights. Control experiments in the small intestine, liver, and spleen showed that AST-NPs protected the whole body from abdominal radiation-induced injury. Notably, AST-NPs also mitigated radiation-induced damage in the duodenum and jejunum [157].

AST also plays a role in the prevention and treatment of non-alcoholic fatty liver disease (NAFLD) [131]. NAFLD represents a form of hepatic injury triggered by metabolic stress, which is intricately associated with insulin resistance and genetic predisposition. Current studies have shown [132–135] that NAFLD can also be caused by obesity, type 2 diabetes [136–138], and dyslipidemia [139–141]. Considering obesity factors [142–145], researchers investigated the potential of AST to reduce obesity-related metabolic imbalance, inflammation, and fibrosis in NAFLD animal models. Their results demonstrated that AST improved the fat-burning capacity of mitochondrial skeletal muscle in mice by attenuating hepatic and adipose tissue inflammation and fibrogenesis [146]. In addition, the incidence of obesity was reduced. The dual-targeted AST-NPs (AXT@TWG@LBGs) designed by Zhang et al. [147] effectively reduced insulin resistance, lowered the levels of inflammatory factors, and alleviated lipid metabolism disorder. The preparation process of AXT@TWG@LBGs involved 3 steps: First, AST was encapsulated within whey protein-based nanoparticles; second, bacterial shells were prepared; and finally, the AST-loaded whey protein nanoparticles were combined with the bacterial cell shells. The orally administered AXT@TWG@LBGs withstood digestion in the gastrointestinal tract and effectively improved the utilization rate of AST (Fig. 4A). The nutritional intervention of AXT@TWG@LBGs was also evaluated in non-alcoholic steatohepatitis mice by assessing liver morphology and performing hematoxylin and eosin staining and oil red O staining of dry tissue sections. The results showed that intervention with AXT@TWG@LBGs effectively inhibited the accumulation and degeneration of liver fat. Additionally, Masson staining revealed a significant reduction in collagen deposition in liver tissues following treatment, indicating slowed fibrosis progression (Fig. 4B, I to IV). Overall, these nanoparticles act directly on stem cells and fibrotic areas while indirectly alleviating liver inflammation by regulating intestinal microbiota and enhancing barrier function, providing a multi-faceted and intelligent therapeutic strategy for non-alcoholic steatohepatitis.

#### Kidney

Several studies have reported a close link between severe burns and acute kidney injury (AKI). Severe burn patients release a large number of inflammatory mediators after burns, which leads to microcirculation disorders and renal vasoconstriction. These conditions directly damage renal tubular epithelial cells, slow renal blood flow, and decrease glomerular filtration rate, resulting in AKI. Guo et al. [148] reported that AST suppressed the expression of pro-inflammatory mediators, including myeloperoxidase, IL-1β, and IL-6, inhibited the activation of the TLR4/MyD88/NF-κB signaling pathway, and increased the renal expression of HO-1. These responses helped the kidneys regulate oxidative stress and the TLR4 pathway, thereby inhibiting the progression of AKI. Moreover, Naito et al. [149] found that AST normalized creatinine and uric acid levels, reduced urea content in diabetic rats, and inhibited glomerular hypertrophy, thereby improving renal function. Moreover, AST up-regulated the expression of antioxidant enzymes, preserved the antioxidant capacity of the parenchyma and plasma, and effectively reduced renal complications caused by diabetes [150,151]. In addition, AST plays an important role in the prevention of renal fibrosis by reducing the accumulation of ECM components and activating the transcription factor Nrf2-ARE [151]. To address the inadequate solubility and stability of AST, liposome-encapsulated AST drug delivery systems were used to target diabetic nephropathy (DN). Glucose transporter 1 is essential in the transport of glucose to glomerular mesenchymal cells. Therefore, glucose-modified liposomes loaded with AST entered cells smoothly with the help of glucose transporter 1 on the glomerular mesenchymal cell membrane [152], facilitating the efficient removal of intracellular ROS generated by oxidative stress.

Chen et al. [153] designed a kidney-targeting nano-delivery system based on AST (AST-GLU-LIP) for the treatment of DN. This design involved preparing glucose ligands to modify AST-loaded liposomes, which then targeted the renal glomerular mesangium based on glucose transporter type 1 (GLUT1) on the cell membrane. Electron microscopy revealed that AST-GLU-LIP nanoparticles exhibited a spherical or elliptical morphology. By observing the clearance of H_2_O_2_ and the apoptosis level of human renal mesangial cells (HRMCs), AST-GLU-LIP exhibited an efficient antioxidant effect and effectively reduced the apoptosis of HRMCs induced by oxidative stress. In addition, the expression of GLUT1 was assessed in the renal tissues of DN rats using an immunofluorescence assay by labeling AST-GLU-LIP with free 1,1′-dioctadecyl-3,3,3′,3′-tetramethylindotricarbocyanine iodide fluorescence. The results showed that AST-GLU-LIP, through modification of the glucose ligand, showed enhanced enrichment in the kidneys and significantly down-regulated the expression of GLUT1. Kisaoglu et al. [154] investigated the effects of different doses of AST on oxidative damage in rats with renal ischemia–reperfusion injury and concluded that AST scavenged free radicals and safeguarded cells and tissues against oxidative injury. Immunoreactivity analysis of Beclin-1, LC3b, and p62 proteins in the high-dose AST group showed that renal protection was mediated through the induction of autophagy. In addition, the uptake of AST-loaded liposomes by endothelial cells was confirmed using Raman spectroscopy and microscopy.

#### Intestine

As a chronic inflammatory intestinal disease, ulcerative colitis is characterized by excessive oxidative stress caused by the accumulation of neutrophils and activation of proinflammatory transcription factors and cytokines, along with increased permeability of the epithelial crypts and intestinal mucosa. Studies showed that AST suppressed oxidative stress-mediated inflammation by increasing the levels of glutathione peroxidase, SOD, and catalase, which reduced the production of ROS and lipid peroxides. Yu et al. [52] demonstrated the clinical applicability of AST-loaded protein-based nanoparticles in the treatment of inflammatory bowel disease. Using molecular dynamics simulations and experimental screening, WPI was identified as the optimal carrier. Based on the natural pH sensitivity and mucosal adhesion properties of WPI, stable AST-loaded nanoparticles were formed via emulsion solvent evaporation, without the need for a crosslinking agent. In this process, WPI and AST were dissolved in an aqueous phase, an oil-in-water emulsion was formed by emulsification technology, and the organic solvent was evaporated under reduced pressure. The protein and AST were precipitated together to form AST-WPI nanoparticles. AST significantly reduced oxidative damage by scavenging ROS, inhibited the expression of inflammatory factors (such as interferon-γ pathway-related proteins), and improved intestinal immune balance.

In addition, AST was effective in treating necrotizing enterocolitis in preterm infants. In a rat experimental model of necrotizing enterocolitis [155], AST reduced the intestinal damage caused by oxidative stress, inflammation, and apoptosis. Simultaneously, a number of investigations have reported a correlation between chronic inflammation/oxidative stress and colorectal cancer (CRC). Obesity-associated metabolic disorders, encompassing excessive oxidative stress and low-grade chronic inflammation, are linked to the progression of CRC. Kochi et al. [156] used type 2 diabetic mice as an animal model and found that AST significantly inhibited the activity of NF-κB and cell proliferation in the colonic mucosa of mice and reduced the expression of IL-1β and IL-6, thereby inhibiting the development of precancerous lesions of colon cancer. In addition, Zhang et al. [157] designed a microalgae nano-integrated system (SP@ASXnano) to reduce the intestinal and systemic damage caused by radiation. Pathological examination revealed that the intestinal crypts were severely damaged by radiation and could not regenerate. In the experimental group supplemented with SP@ASXnano, regenerative cells in the intestinal crypts of the duodenum, jejunum, and ileum were effectively protected. In addition, it was found that radiation damaged the integrity of the small intestinal epithelium, causing hyaline degeneration of stem cells, and resulting in a significant reduction of lymphocytes in the spleen, with an unclear boundary between the red pulp and the white pulp. In contrast, through intragastric treatment with SP@ASXnano, the histological structure of these organs was maintained (Fig. 4C). In summary, SP@ASXnano integrated microalgae can resist radiation damage by maintaining the integrity of the intestinal villi structure, reducing pro-inflammatory factors, and enhancing endogenous antioxidant defense.

### Other applications

#### Medical dressings

Bioactive microparticles can promote wound healing and offer excellent biocompatibility, antibacterial and anti-infective functions, superior mechanical properties, and maneuverability. AST is an ideal additive for novel functional wound dressings owing to its antioxidant, antibacterial, anti-inflammatory, and pro-tissue repair properties. Monavari et al. [55] reported that hydrogel structures containing bioactive microparticles and AST could facilitate stable and sustained delivery of AST. Moreover, 3D printing technology enables personalized and precision treatment, showing great potential in treating chronic wounds, burns, and surgical incisions, and is expected to become an important tool for wound repair and tissue regeneration. In addition, Mussagy et al. [158] combined AST with natural rubber latex (NRL) to develop a functional dressing. AST was first encapsulated in polymer micelles and then integrated into NRL bandages, leveraging the antioxidant, anti-inflammatory, and pro-healing properties of AST and the flexibility and biocompatibility of NRL. The dressing has been applied to treat chronic wounds, burns, postoperative damage, sports injuries, and other conditions. In addition, diabetic patients face a lifelong risk of persistent nonhealing wounds, such as diabetic foot ulcers, largely due to excessive generation of ROS. Kang et al. [159] designed a hydrogel dressing based on microalgae (HEA@Gel) that delivered soluble oxygen and AST to the wound to trigger in situ light curing. This multifunctional approach promoted the rapid healing of diabetic wounds in a comprehensive manner by leveraging the antibacterial, oxygen-releasing, ROS-reducing, and anti-inflammatory effects of AST. Characterization of HEA@Gel demonstrated that the hydrogel effectively promoted cell proliferation and migration, providing an advanced strategy to improve the therapeutic effect of AST.

#### Cancer treatment

Recent studies have shown that AST has a significant inhibitory effect against malignant tumors, such as oral cancer [160], bladder cancer, CRC [32], leukemia, and hepatocellular carcinoma [126,127]. Its anti-cancer mechanisms mainly involve 6 pathways: Inhibiting proliferation, inducing apoptosis, modulating oxidative stress, promoting anti-inflammatory responses, blocking invasion and metastasis, and regulating intercellular communication. To inhibit tumor proliferation, AST interferes with the growth factor signal transduction and MAPK pathways [161]. Animal model experiments confirmed that AST inhibited the proliferation of CBRH-7919, SHZ-88, and Lewis cancer cells, with no significant effect on normal hepatocyte HL-7702 cells [127], demonstrating its targeted therapeutic properties.

In terms of the apoptotic mechanism, AST promotes programmed cell death of cancer cells by activating the mitochondrial pathway, which provides a theoretical basis for reducing tumor volume and improving prognosis. In view of the cancer characteristics of oxidative imbalance, studies have shown that AST enhances antioxidant defense and inhibits K562 leukemia cell cycle progression through the Nrf2 pathway; however, the specific role of ROS signaling in the anticancer effect of AST still needs further exploration.

As for the mechanism correlating inflammation and carcinogenesis, AST can effectively inhibit the release of pro-cancer factors, such as IL-6 and TNF-α, and block the proliferation stimulation and immune escape mediated by inflammation [162]. During tumor invasion and metastasis, AST significantly down-regulates the mRNA and protein expression of MMP-2/9 and inhibits ECM degradation by inhibiting the functionality of MMP. Notably, these mechanisms have dose-dependent and tissue-specific differences, and the precise molecular targets and regulatory dynamics of the associated signaling network require further systematic investigation. Lu et al. [117] developed nanoparticles (LA/DOX NPs) containing azithromycin, low-molecular-weight heparin (LMWH), and AST, which inhibited liver and lung metastasis of breast cancer. First, the hydrophilic LMWH and the hydrophobic AST were linked through ester bonds to form an LMWH-AST conjugate. Then, this conjugate was placed in water for self-assembly to obtain LA NPs. The activation of NF-κB and signal transducer and activator of transcription 3 (STAT3) in cancer cells leads to the production of a large number of downstream inflammatory factors, which in turn promote the development of the tumor and the recruitment of inflammatory cells, including myeloid-derived suppressor cells, neutrophils, and macrophages. These recruited inflammatory cells further sustain the activation of inflammatory signaling pathways, resulting in the recruitment of more inflammatory cells by the released cytokines. Through this vicious cycle, the inflammatory response within the tumor further intensifies, accelerating the development of breast cancer and its metastasis to the liver and lungs. After treating 4T1 tumor-bearing mice with phosphate-buffered saline and LA/DOX NPs for 40 d, hematoxylin and eosin staining showed that the livers and lungs of the mice in the phosphate-buffered saline group had obvious metastatic nodules, while those in the LA/DOX NP group did not show any metastatic nodules and were similar to those of normal mice. Furthermore, immunohistochemical analysis of tumor tissues showed that the number of Ki67-positive cells and the expression of MMP-9 in the LA/DOX NP group were both decreased, confirming that LA/DOX NPs promoted apoptosis of tumor cells.

## Summary, Outlook, and Challenges

AST, as a naturally occurring ketone-type carotenoid, has attracted significant attention in the biomedical field in recent years owing to its outstanding antioxidant, anti-inflammatory, and immune-regulating properties. Its molecular structure contains conjugated double bonds and hydroxyl/ketone groups, which provide it with extremely strong free radical scavenging ability, effectively inhibiting oxidative stress, reducing inflammatory responses, and playing a crucial role in regulating cell signaling pathways. These characteristics highlight the great application potential of AST in various fields such as tissue engineering, drug delivery systems, wound healing, antitumor treatment, neuroprotection, and cardiovascular disease intervention. In the field of biomaterials, AST has been successfully integrated into various carrier systems, such as polymer nanoparticles, liposomes, hydrogels, electrospun fibers, and 3D-printed scaffolds. These carriers not only improve the stability and bioavailability of AST but also endow it with controlled release and targeted delivery capabilities. For example, in skin tissue engineering, AST-loaded hydrogel dressings significantly promote collagen deposition, angiogenesis, and epithelial regeneration while reducing oxidative damage and scar formation; in myocardial repair, AST-modified scaffolds alleviate ischemia–reperfusion injury and improve cardiac function. Additionally, in tumor treatment, AST inhibits cancer cell proliferation by regulating pathways such as NF-κB, Nrf2, and PI3K/Akt, and enhances the sensitivity of chemotherapy drugs. Moreover, the neuroprotective effect of AST shows promising prospects in the intervention of neurodegenerative diseases such as Alzheimer’s and Parkinson’s disease.

Although significant progress has been made in the research of bio-materials based on AST, its clinical application still faces many challenges that need to be overcome. First, the strong hydrophobicity of AST greatly limits its solubility and stability in the physiological environment. Therefore, it is necessary to optimize delivery systems by developing new nanocarriers such as exosomes, metal–organic frameworks, and biomimetic nanoparticles to enhance targeting and achieve sustained-release performance. Moreover, designing a stimulus-reactive release system (such as related to pH value, ROS, or enzyme trigger mechanisms) is warranted to achieve precise accumulation at the lesion site. Second, long-term biocompatibility, in vivo metabolic pathways, and potential toxicity, especially when AST is used at high doses or for a long time, require systematic assessment. It is recommended to combine advanced models with multi-omics technologies to deeply analyze pharmacological and toxicological mechanisms. Finally, most of the current research is conducted at the in vitro or animal experimentation stage. In the future, it is necessary to promote standardized clinical trials to verify the efficacy of AST and develop low-cost, scalable production processes to accelerate the industrialization process. In addition, exploring the synergistic effects of AST and growth factors, small interfering RNA (siRNA), immunomodulators, and other active molecules, and constructing smart “integrated diagnosis and treatment” materials is an important research direction. For example, photothermal therapy or immune checkpoint inhibitors could be combined to enhance antitumor effects, or neurotrophic factors could be coordinated to optimize the neural regeneration microenvironment. Despite these challenges, AST has broad application potential in emerging fields such as gene editing, regulation of the intestinal flora, anti-aging, and eye diseases.

In conclusion, significant progress has been made in the research of loading AST onto biomaterials, but many challenges remain. This article reviewed the types, preparation methods, and advantages of biomaterials containing AST and summarized their applications in tissue engineering and drug delivery. These findings lay the foundation for future research on biomaterials loaded with AST.

## Data Availability

No data were used for the research described in the article.
